# Three dimensional microcarrier system in mesenchymal stem cell culture: a systematic review

**DOI:** 10.1186/s13578-020-00438-8

**Published:** 2020-06-03

**Authors:** Benson Koh, Nadiah Sulaiman, Mh Busra Fauzi, Jia Xian Law, Min Hwei Ng, Ruszymah Bt Hj Idrus, Muhammad Dain Yazid

**Affiliations:** 1 Tissue Engineering Centre, Universiti Kebangsaan Malaysia Medical Centre, Jalan Yaacob Latif, Cheras, 56000 Kuala Lumpur, Malaysia; 2 Department of Physiology, Faculty of Medicine, Universiti Kebangsaan Malaysia Medical Centre, Jalan Yaacob Latif, Cheras, 56000 Kuala Lumpur, Malaysia

## Abstract

Stem cell-based regenerative medicine is a promising approach for tissue reconstruction. However, a large number of cells are needed in a typical clinical study, where conventional monolayer cultures might pose a limitation for scale-up. The purpose of this review was to systematically assess the application of microcarriers in Mesenchymal Stem Cell cultures. A comprehensive search was conducted in Medline via Ebscohost, Pubmed, and Scopus, and relevant studies published between 2015 and 2019 were selected. The literature search identified 53 related studies, but only 14 articles met the inclusion criteria. These include 7 utilised commercially available microcarriers, while the rest were formulated based on different surface characteristics, all of which are discussed in this review. Current applications of microcarriers were focused on MSC expansion and induction of MSCs into different lineages. These studies demonstrated that MSCs could proliferate in a microcarrier culture system in-fold compared to monolayer cultures, and the culture system could simulate a three-dimensional environment which induces cell differentiation. However, detailed studies are still required before this system were to be adapted into the scale of GMP manufacturing.

## Introduction

### Mesenchymal stem cells

Adult mesenchymal stem cells are becoming increasingly popular as a potential cell source in regenerative medicine nowadays. This multipotent CD 34^−^ fibroblast-like stem cell has the ability to differentiate into specialized cells such as adipocytes, osteocytes, chondrocytes, and myocytes [1–3]. It can be isolated from various adult tissue sources such as blood or adipose tissue, dermis, muscle, dental pulp, and Wharton’s jelly [4–7]. In contrast to embryonic pluripotent stem cells, MSC is devoid of ethical, histocompatibility, and teratomas-formation issues. In addition to that, several studies successfully demonstrated the efficacy of MSCs in regenerating new tissues and repair defects [8–11].

Stem cell-based regenerative medicine is an emerging approach for tissue reconstruction. Allogenic hematopoietic stem cell transplant has the potential to play a significant role in the treatment of autoimmune diseases or hematopoietic disorders. However, the applications of therapy are limited due to morbidity and mortality of graft versus host disease (GVHD). Studies have reported that mesenchymal stem cells could reduce inflammatory cytokines through interplay with several subsets of immune cells; thus the immunoregulatory capacity of MSCs makes them of great interest in clinical studies involving GHVD [12–14].

### Anti-inflammatory properties of mesenchymal stem cells

Aside from its regenerative capabilities, MSCs are known for its immunosuppression or anti-inflammatory ability in cell transplantations. The role of MSCs as an anti-inflammatory agent has become more evident with the elucidation of the mechanism of inflammation, which includes the release of intracellular cytokines such as interleukin-1α from injured cells or activation of macrophages by pathogen-associated molecular patterns (PAMPs) or damage-associated molecular patterns (DAMPs) interaction with receptors to generate proinflammatory cytokines [15–17].

According to the results reported by [18], administration of MSCs into a mouse model successfully inhibited bleomycin (BLM)-induced elevation of TNF-α, IL-1α, and IL1RN mRNA in the lungs, which protected lung tissues from BLM-induced injury by blocking TNF-α and IL-1α, the main proinflammatory cytokines in the lungs. A similar anti-inflammatory property was reported by Oh et al., where the suppression of IL-2 and IFN-γ, and the reduced infiltration of CD4^+^ cells by MSCs, showed a reduction in corneal inflammation and neovascularisation [19]. In short, the anti-inflammatory effects of MSCs have been reported in various events such as lung injury, myocardial infarction, corneal injury, sepsis, and diabetic wound healing [20–23].

### Cytokines in inflammatory events

Inflammatory mechanisms in GVHD were generally associated with activation of immune cells (T cells, B cells, and macrophages) in the presence of antigen-presenting cells (APC). These immune cells will release substances called cytokines which regulate or facilitate immune responses. For instance, the IL-1 pathway plays a crucial role in generating sterile inflammation, which is similar in effect as that produced by tumor necrosis factor-α (TNF-α) in lung injuries [24]. In addition, the presence of pro-inflammatory cytokines such as TNF-α and IL-6 in serum also contributed to sepsis in a mouse model [25]. In addition to that, the secretion of TNF-α and IL-1α by macrophages also induced peritonitis in a mouse model [26].

TNF-α is a prototypical member of a large superfamily known as TNF/TNFR superfamily, which comprises more than 40 family members. The TNF-α gene is a single-copy gene on human chromosome 6 (murine chromosome 17), which codes for a 27-kDa (233 amino acid) protein that is proteolytically cleaved into a 17-kDa (157 amino acid) molecule [27]. TNF-α is secreted from activated macrophages by induction of Toll-like receptors and other factors, and generally after priming with interferon gamma (IFN-γ). It is rapidly released after trauma, infection, or exposure to bacterial-LPS and was shown to be one of the early abundant mediators in inflamed tissues. Apart from that, the role of TNF-α during inflammation is mostly associated with coordination of the pro-inflammatory cytokine cascade. Therefore, TNF-α is considered as a master regulator of pro-inflammatory cytokines during inflammation [28].

### Mesenchymal stem cells expansion

Clinical applications of mesenchymal stem cells require billions of cells [29] and two-dimensional platforms, which might pose a challenge in scaling-up. In a clinical study of acute ischemic stroke, it was suggested that the number of MSCs required for administration to a single patient ranged from 1–8 × 10^6^ MSCs per kg of body mass, depending on the indication [30]. Innovation of cell culture products aim to address surface limitations imposed by monolayer culture flasks. Multi-layered flasks which could accommodate up to 40 layers of culture chambers is a good example of such innovation. However, difficulty in observing the in-cultured cells could be a potential downside of this innovation. In order to achieve a scalable undifferentiated mesenchymal stem cell number for cell transplantation and tissue engineering applications, 3-dimension culture techniques seem to be a more reliable approach compared to 2D cultures. Mesenchymal stem cell expansion in bioreactors potentially provide ease of scalability, flexible modes of operation, better process monitoring, and control compatibility. For example, Zhou et al. (2013) developed a novel strategy for 3D expansion of bone marrow MSCs, which produced a 10.4 ± 0.8-fold increase compared to 2D cultures on day 5.

### 3-D cell culture

Various tissue-engineering studies utilising a 3D scaffold system have shown their efficacy in in vitro culture of MSCs. Three-dimensional culture conditions simulates environment of cells in vivo, therefore providing a suitable condition that enhances cellular activities that are not observed in normal monolayer cultures [31].

While 3D scaffold systems propose unique attractive advantages, these also brought about significant challenges for MSC culture including: (i) the use of undefined components from human or animal tissue, which may result in batch-to-batch variation and poses risks for pathogen and immunogen transfer [32, 33], and thus an obstacle for good manufacturing practice (GMP) in cell production [34]; (ii) substantial cell aggregation that could possibly lead to MSC differentiation or senescence [35]; (iv) limited cell expansion rates and yield per volume [36]; and (v) unpredictable consequences of long-term serial expansion.

One way to address a few of the abovementioned challenges is to adapt the use of microcarriers. These micron-sized spherical particles were initially used for the growth of adherent cells for viruses and production of vaccines [37–39]. Over the decades, properties of microcarriers underwent various modification and innovation to meet the need of different cell types. To date, there are numerous manufacturers and multiple microcarrier varieties are commercially available.

### Microcarrier in 3-D culture

Microcarriers provide surface matrices that enable attachment of adherent cells to form cell-microcarrier complexes suspended in growth medium [40]. The fundamental structure of microcarriers are tiny beads (size ranging from 100–300 microns) that are able to maintain suspension during stirring. A number of microcarriers have been synthesized and made commercially available, e.g. glass, diethylaminoethyl (DEAE)-dextran, acrylamide, polystyrene, collagen, and alginate [41].

**Fig. 1:**
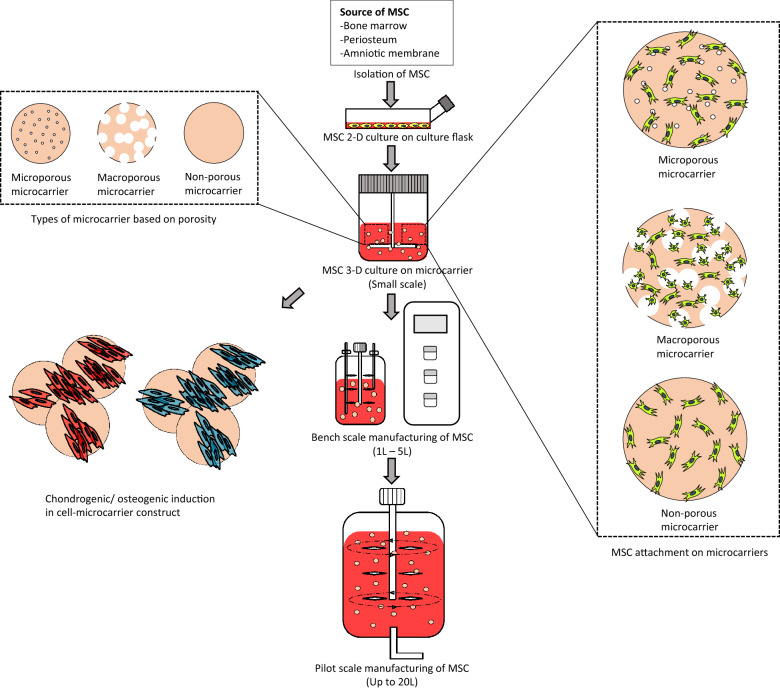
Schematic illustrating the basic flow of up-scaling MSC culture from laboratory scale into manufacturing scale. To date, a “pre-adaptation” period prior microcarrier culture system in MSC is still required, where 2-D culture flasks were used for cell isolation. The up-scale of MSC production can be first optimising culture condition in a small-scale culture system (usually 10–500 mL), followed by up-scaling into bench scale (1–5 L), and finally up to manufacturing scale (up to 20 L). There are 3 major types of microcarrier: non-porous, microporous and macroporous. Cells attach differently based on the porosity of the microcarrier. In general, cells will be attached on the surface of non-porous and microporous microcarrier; while microporous microcarrier provides larger spaces, which allow cells to attach into the inner part of the microcarrier. Due to the similarity towards human body environment, MSCs-microcarriers constructs were found to be able to differentiated into osteo- and chondro-lineage in a specific condition

Microcarrier-based cell culture systems are relatively flexible as they promote higher cell yield and can be integrated into existing bioprocess manufacturing systems such as stirred bioreactors and spinner flasks [42]. Such microcarriers have been established for vaccine production or fermentation processes decades ago, however, downstream processes were only focused on metabolites instead of cells. In cell-based therapy, the product of interest are the cells itself, and the main objective of bioprocessing changed from maximising the yield of metabolites to harvesting large quantities of MSCs. Since mesenchymal stem cells required a support surface for cell division, microcarriers are often added into culture media to provide sufficient adherent surface for MSCs in three-dimensional culture. Figure 1 shows the basic approach of up-scaling MSC production in microcarrier-based culture system. Microcarriers provide a large surface area for cell growth during proliferation in suspension cultures, thus allowing scaling-up of cell production in small volumes of medium [43]. In addition to that, the suspended system provides better nutrient intake and gas exchange, and at the same time the adjustable stirring mechanism provides control over shear stress which might facilitate differentiation along certain lineages [44]. This approach could be an ideal model for MSC expansion for its large surface area per unit volume of media compared to T-flask cultures. Hence, the selection of microcarriers are crucial as it would contribute a direct impact on cell expansion.

To date, there are vast reports which suggests extensive choices of suitable microcarriers for mesenchymal stem cell culture. Alginate/PEG-based microcarriers could provide good attachment and proliferation of human umbilical cord blood mesenchymal stem cells, with well-controlled microcarrier degradation for harvesting [45]. The use of Cytodex type 1 from GE healthcare for porcine bone marrow-derived MSCs could produce cell numbers of approximately 4 × 10^5^ cells/mL [46], while the use of Cytodex type 3 showed similar cell numbers (3.8 × 10^5^ cells/mL) for human placental MSCs [47].

## Methods

### Search strategy

The review was conducted to systematically assess articles on the application of microcarriers for MSC culture. Three databases were comprehensively used to search for relevant studies; Medline via Ebscohost, Pubmed, and Scopus. The keywords used were the combination of words “Mesenchymal Stem Cell” AND “Microcarriers”.

### Selection criteria

The year limit for searches was from 2015 to 2018, and only studies published in English were considered. The search outcomes identified all articles containing the word “mesenchymal stem cell” and “microcarrier”. Databases were searched individually to ensure all relevant studies were considered. The titles and abstract were carefully screened for eligibility related to the topic of interest. Primary studies related to microcarrier application were included. Review articles, news articles, letters, editorials, and case studies were excluded from the search.

### Data extraction and management

Data were extracted from each eligible article by two reviewers. The selected papers were screened in several phases prior to inclusion. First, titles that were not relevant to the topic were excluded. Next, abstracts of the papers were screened, and unrelated studies were excluded. All duplicates were removed. The following data were summarized from the selected studies: authors, year, source of MSCs, applications, type of microcarrier used, results, and conclusion.

## Results

### Search result

**Fig. 2:**
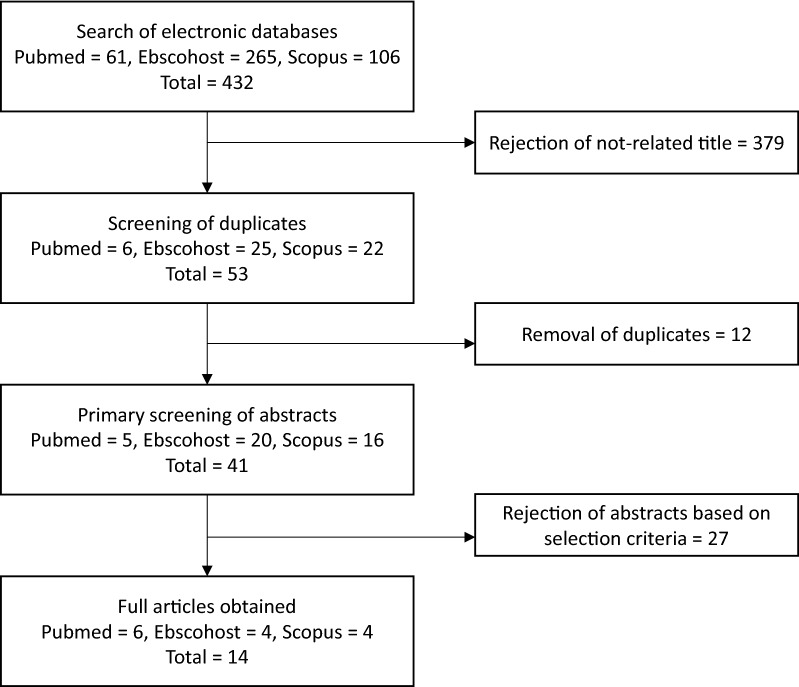
Flow chart of the article selection process from Pubmed, Ebscohost, and Scopus databases

The primary search identified 432 articles: 61 articles were derived from Pubmed, 265 from Ebscohost, and 106 articles from Scopus. To minimize bias and improve the strength of the related articles, two reviewers independently assessed the articles according to the inclusion and exclusion criteria. A total of 379 articles were removed as they were unrelated to either mesenchymal stem cells or microcarriers. A joint discussion was conducted to achieve consensus on differences which emerged during the assessment. From the 53 remaining articles, 12 duplicates were removed before full articles were retrieved. From the remaining 41 articles, 27 articles were rejected based on the inclusion criteria as these articles were not primary studies, were not related to mesenchymal stem cells or microcarriers, or were unavailable as full articles. Finally, a total 14 studies were selected for data extraction in this review. The flow chart of the selection process is shown in Fig. 2.

### Study characteristics

All studies were published between 2015 and 2019 and reported on in vitro studies. Thirteen studies utilised human mesenchymal stem cells, while only one reported using rat MSCs. Seven out of 14 articles utilised commercially available microcarriers, while the rest were formulated based on different surface characteristics. From the generated data, articles were classified into three aspects: Microcarriers in MSC culture, MSC expansion and MSC differentiation. A summary of the studies is provided in Table 1.

**Table 1:** Summary and classification of the 14 articles selected from the database search

	Author	Sample source	Application	Microcarrier used	Culture media used	Result	Conclusion
1	Chui et al. [51]	Human MSCs-hTERT cell line	MSC expansion	Electrosprayed genipin cross-linked alginate-chitosan microcarrier Cytodex 1	Low glucose DMEM + 10% FBS	MSCs cultured on fabricated microcarriers had a 26% higher cell attachment and twice the proliferation rate compared to the commercial microcarrier No significant difference in gene expression between the two microcarriers for the positive MSC surface markers as well as showing either low or no signal for negative MSC surface markers	Genipin cross- linked alginate–chitosan based microcarriers can act as a potential alternative to commercial microcarriers for MSC expansion
2	Gupta et al. [60]	Human periosteum-derived cells	MSC Bone forming potential	Cultispher S	High glucose DMEM + 10% FBS/HPL	HPL resulted in faster cell proliferation compared to FBS Cell viability and trilineage differentiation capability were that maintained by HPL, although a suppression of adipogenic differentiation potential was observed HPL supplementation resulting in almost three times more mineralized tissue within calcium phosphate scaffolds	The use of HPL in bioreactor-based expansion of hPDCs is an optimal solution that increases expansion efficiency along with promoting bone forming capacity of these cells
3	Krutty et al. [59]	Bone marrow MSC	MSC expansion	PNIPAM grafted microcarriers	MEM α + 10% FBS	The microcarriers create a reproducible surface that does not rely on the adsorption of xenogenic serum proteins to mediate cell adhesion MSCs cultured on this fabricated microcarriers achieve sixfold expansion and retain their ability to differentiate after harvesting	PNIPAM grafted microcarriers are a relevant platform for expanding cells while maintaining hMSC functionality
4	Tanimowo Aiyelabegan et al. [57]	Rat bone marrow MSC	MSC osteogenic differentiation	k-casein conjugated agarose microspheres	DMEM	The cell viability of the synthesized microspheres significantly different from uncoated microspheres, but similar to the control and commercial microcarriers This microcarrier systems upregulated the expression of osteo- genic differentiation markers on bone marrow mesenchymal stem cells cultured on the carrier systems	k-casein conjugated agarose microspheres culturing environment may assist in reducing the need for expensive hormones and growth factors that directs differentiation, and thus, could reduce the risk of unwanted systemic side effects in vivo and aid the clinical translatability of MSCs that are cultured using this strategy for bone TE
5	Heathman et al. [63]	Bone marrow MSC	MSC expansion	Plastic P-102L microcarriers	PRIME-XV MSC Expansion SFM	It was found that growth rate though an intermediate value of ~ 1.3 NJS did not cause sampling difficulties, clumping and poor growth. At this range of agitation intensities, cell quality remained unchanged post-harvest Direct aeration of the culture medium both with and without Pluronic F68 via a sparger at NJS was detrimental to BM-hMSC growth	Alternative methods of supplying sufficient levels of oxygen to microcarrier bioreactor systems culturing BM-hMSCs may have to be developed as well as establishing the level of pCO2 that they can tolerate as these systems are scaled up to manufacture commercially-viable cell numbers
6	Yuan et al. [62]	Bone marrow MSC	MSC expansion	PNIPAM grafted microcarriers	MEM α + 10% FBS	hMSC aggregates generated from the bioreactor maintained comparable immunomodulation and cytokine secretion properties compared to the ones made from the culture plate At room temperature, hMSCs were self-assembled into 3D hMSC aggregates in PBS-VW bioreactor and remain as single cells in bioreactor owing to different hydrodynamic conditions	Thermal responsive microcarriers could scale-up the production of hMSC aggregates in the suspension bioreactor for integrated cell expansion
7	Dias et al. [58]	hMSC (Lonza, Walkersville, MD)	Serum-free MSC expansion	PEG-based hydrogel coated Hillex II amine-functionalized microcarriers	MEM α + 10% FBS Lonza serum-free MSC growth media	High cell expansion was apparent in serum-free media on coated microcarriers with some aggregation during expansion Osteoblast and adipocytes differentiation apparent in serum-free condition on PEG	The PEG hydrogel coating reduced microcarrier aggregation during MSC culture
8	Lin et al. [69]	Fetal bone marrow MSC	hMSC-microcarrier constructs chondrogenic differentiation	Cytodex 1 Cytodex 3 SphereCol Cultispher-S	MEM α + 10% FBS	Narrow range of 70% cell confluency, cell number of 10 x 10^3, and microcarrier of 300 per construct generate the optimal microenvironment for efficient chondrogenic differentiation	Scalable microcarrier-spinner cultures enhance the chondrogenic potential of the MSC, supporting their use for large-scale cell expansion in cartilage cell therapy
9	Rafiq et al. [61]	Bone marrow MSC (Lonza, Walkersville, MD)	Automated hMSC expansion	Plastic P102-L microcarrier	DMEM + 10% FBS PRIME-XV MSC Expansion SFM	More than 250% increase in yield compared to the serum-based process The combination of both serum-free and automated processing improved the reproducibility more than tenfold compared to the serum-based, manual spinner flask process	Ambr15 microbioreactor is an effective tool for bioprocess development of hMSC microcarrier cultures and improves both process yield and consistency.
10	Takahashi et al. [70]	Bone marrow MSC	MSC expansion	Cytodex 1	Low glucose DMEM + 10% FCS	30 rpm was the lowest agitation rate necessary for the suspension of Cytodex 1 microcarriers, and the cells grew fastest at 60 rpm The percentages of CD90- and CD166-positive cells among cells grown on Cytodex 1 at 60 rpm (91.5 and 87.6%) were comparable to those of cells grown in the pre-culture on dishes	Beads-to-beads subcultivation method maintaining the expressions of the cell surface antigens CD90 and CD166, while adjusting agitation rate could decrease the microcarrier aggregation
11	Zhang et al. [71]	Human amniotic MSC HUVEC	Pre-vascularized modular bone tissue fabrication	CultiSpher S	DMEM + 10% FBS MEM α + 10% FBS	Microtissues were formed with high cellularity after 4 weeks culture in spinner flask, evenly distributed cells and tube formation ability Coculture with HUVECs exerted an inhibitory effect on osteogenic differentiation of MSCs	An effective method to fabricate pre-vascularized bone microtissues was established, which would lay a solid foundation for subsequent development of vascularized tissue grafts for bone regeneration
12	Nienow et al. [54]	Bone marrow MSC	MSC expansion	Solohill plastic Solohill collagen	DMEM + 10% FBS	hMSCs were successfully cultured using the minimum agitator speed required for complete microcarrier suspension The cells were shown to retain their desired quality attributes and were able to proliferate with the reported cell detachment protocol	Theagitation strategy with respect to culture and harvest therefore offers a sound basis for a wide range of scales of operation
13	Song et al.[56]	Bone marrow MSC	MSC expansion	Thermosensitive glass microcarrier	Low glucose DMEM + 10% FBS	NIPAAm was successfully grafted on to the surface of the microcarriers, providing an excellent biocompatible environment for BMMSC adhesion and growth BMMSCs could be fully removed from the thermosensitive glass microcarriers with remained cell viability	This new substrate can provide a better 3D environment for cell growth and cell recovery, which is expected to be utilized in vitro for massive cell expansion by combining with the dynamic bioreactor
14	Lakhkar et al. [55]	hMSCs MG63 osteoblast-type cells	MSC Oesteogenic induction	Titanium phosphate glass microcarrier	Low glucose DMEM + 10% FCS	The microcarrier proliferative capacity is increasing in MG63 cell Expression of bone morphogenetic protein-2 and osteopontin, significantly greater Scanning electron microscopy and confocal laser scanning microscopy images reveal favorable MG63 and human mesenchymal stem cell adhesion on the Ti5 microsphere surfaces	The titanium phosphate glass microcarrier function as platforms for guided osteogenic differentiation of hMSCs. It is expected that these approaches will in future facilitate the development of viable bone tissue in vitro for use in bone replacement therapies

## Discussion

The database search provided 14 articles related to Wharton’s Jelly and microcarrier. From these articles, various sources were examined regarding microcarrier application on MSC culture. This review assessed the application of microcarrier on MSC culture, which may have remarkable potential for different usage in future application.

### Microcarrier in MSC culture

Generally, microcarriers can be divided into 3 major types: non-porous, microporous and macroporous (Fig. 1). While non-porous microcarriers are relatively straightforward with limited surface area, the microporous structure of microcarriers allow cells attached on the carrier to undergo material transfer on the basolateral side of the cell; however, the surface area available for cell attachment is also limited on the outer surface of the microcarrier. In contrast, macroporous microcarriers provide a larger pore size that enable cells to enter into the microcarrier. In this case, macroporous microcarriers contributes a larger surface area per millilitre of media compared to microporous microcarriers, hence potentially higher cell yields in large scale cultures [48]. Table 2 shows the summary of the microcarrier used in the 14 studies selected aforementioned, the details were arranged based on the material, surface feature, diameter, porosity, concentration in culture and results of application for each microcarriers.

**Table 2:** Summary and classification of the microcarrier used in the 14 studies selected

Availability	Microcarrier	Material	Surface feature	Diameter (µm)	Porosity	Concentration	Cell yield	Cell differentiation	Manufacturer	Reference
Commercially available	Cell expansion									
	Glass microcarrier (G102-1521)	Cross-linked polystyrene	Silica glass	125–212	Non-porous	35 mg/mL	–	–	Pall corporation	[56]
	Hillex II microcarrier	Polystyrene	Positive charge amine	160–200	Microporous	3 cm^2^ per 24-well	1.2 × 10^5 cells/mL	–	Pall corporation	[58]
	Solohill plastic	Plastic	Plastic surface	90–150	Non-porous	6000 unit/mL	1–5 × 10^5 cells/mL	–	Pall corporation	[54]
						5 cm/mL	8.1 × 10^5 cells/mL^a^	–		[61]
						5 cm/mL	9.6 × 10^4 cells/mL^b^	–		[63]
	Solohill collagen	Polystyrene	Collagen coated	125–212	Non-porous	6000 unit/mL	1–5 × 10^5 cells/mL	–	Pall corporation	[54]
	Cytopores 1	Cellulose	Positive charge (0.9–1.20 meq/g)	230	Macroporous	1.2 mg/mL	1.5 × 10^5 cells/mL	–	GE Healthcare	[70]
	Cytopores 2	Cellulose	Positive charge (1.65–1.95 meq/g)	230	Macroporous	1.2 mg/mL	1.4 × 10^5 cells/mL	–	GE Healthcare	[70]
	Cell expansion & differentiation									
	Cytodex 1	Cross-linked de × tran	Positive charge	147–247	Microporous	2.7 mg/mL	5.2 × 10^5 cells/mL^b^	Chondrogenesis	GE Healthcare	[69]
						3 mg/mL	4.3 × 10^5 cells/mL	–		[70]
						1.7 mg.mL	0.86 × 10^5 cells/mL	–		[51]
	Cytodex 3	Cross-linked dextran	Gelatin coated	141–211	Microporous	4 mg/mL	3.55 × 10^5 cells/mL^b^	Chondrogenesis	GE Healthcare	[69]
						–	–	Osteogenesis		[57]
	CultiSpher S	Gelatine	Gelatine	130–380	Macroporous	0.5 mg/mL	2.46 × 10^5 cells/mL^b^	Chondrogenesis	Sigma	[69]
						2 mg/mL	–	Vascularise bone-like microtissue		[65]
						1 mg/mL	1.3 × 10^5 cells/mL			[60]
	SphereCol	–	Collagen	125–212	Microporous	1.2 × 10^3 microcarrier/mL	3.58 × 10^4 cells/mL^b^	Chondrogenesis	Sigma	[69]
	Silica glass microsphere	Silica	Silica	0.15–5	Non-porous	16 mg/mL	3.3 × 10^5 cells/mL	Osteogenesis	Polysciences Inc	[55]
In-house fabrication/modification	Cell expansion									
	PVG coated microcarrier	Polystyrene	PVG-RGD coated	125–212	Non-porous	–	–	–	–	[59]
	PNIPAM grafted microcarrier	Polystyrene	PNIPAM coated	50–100		10 mg/mL	9.4 × 10^4 cells/mL	–	–	[62]
	Alginate-chitosan microcarrier	Genipin cross-linked-alginate	Chitosan	200–300		–	1.72 × 10^5 cells/mL	–	–	[51]
	PNIPAAm-grafted microcarrier	Cross-linked polystyrene	Thermosensitive PNIPAAm grafted surface	125-212	Non-porous	35 mg/mL	–	–	–	[56]
	PEG coated hydrogel	Polystyrene	PEG hydrogel surface	400	Non-porous	3 cm^2^ per 24-well	1.12 × 10^5 cells/mL	–	–	[58]
	Cell expansion & differentiation									
	Ti5 microcarrier	Titanium phosphate glass	Titanium phosphate glass	50–100	Non-porous	16 mg/mL	4.5 × 10^5 cells/mL	Osteogenesis	–	[55]
	Agarose microcarrier	Agarose	Casein	100–150		–	–	Osteogenesis	–	[57]

^a^Microcarrier added in mid of study

^b^Approximated value

The fabrication material of microcarriers is also a crucial factor in microcarrier cultures because of its physical and chemical effects towards cells, which include porosity, mechanical strength, permeability of nutrients, size, density, and shape [49]. In order to facilitate adherent cells to attach on the carrier surface, the divalent cations or protein available in culture medium is important so that cell could utilise it for attachment. Polymers such as polystyrene, plastic, or glass are commonly utilised as the basic matrix of microcarriers; these microcarriers are usually positively charged or chemically bounded to facilitate the attachment of adherent cells which possess an uneven distribution of negative surface charge. While microcarriers with higher charge densities were developed to promote cell adhesion for cell lines with weak adhesion (E.g. Cytopore 1 & 2), these microcarriers poses a challenge during cell harvesting due to difficulties with cell detachment at the end of the culture [50].

To overcome this problem, biopolymers (dextran, gelatin, cellulose, agarose, alginate) were introduced as they potentially facilitate cell harvesting while providing a biocompatible environment for cultures [51, 52]. In addition, microcarriers with surface modifications (E.g. protein or collagen coated), could also achieve a similar effect as the microcarriers mentioned above. Fibronectin for example, is commonly used to coat plastic or glass microcarriers to increase cell adhesion in microcarrier cultures, and used in concentrations ranging from 1–50 ug/mL [53–55]. On the other hand, compounds such as casein, chitosan, or even PNIPAAm was grafted on the surface of microcarriers to modify its adhesion properties and to provide an easier solution for cell harvesting [51, 56, 57].

### Application of microcarriers in MSC culture

#### MSC expansion

A study found that PEG coated microcarriers supported the expansion of hMSCs in a serum-free environment, with doubling time under 25 h in the growth phase, as well as preserving its osteogenic and adipogenic differentiation post-harvest [58]. Genipin cross-linked alginate-chitosan microcarriers were demonstrated to provide 26% higher MSC attachment and twice the proliferation rate compared to the commercial microcarrier, Cytodex 1. The cells produced were easily detached without an extended incubation period or intense agitation during harvesting [51]. Whereas Krutty et al. developed a chemically defined PVG microcarrier which achieved a six-fold expansion in MSCs, while retaining their ability to differentiate after harvesting [59].

Under xenogenic-free culture conditions, Gupta et al. reported that HPL resulted in faster cell proliferation by 5.2 ± 0.61-fold in comparison to 2.7 ± 02.22-fold in FBS [60]. In addition, an automated serum-free, microcarrier culture system was established. It was found that such approach can produce more than tenfold MSC expansion compared to serum-based, manual spinner flask methods [61].

Several studies have been conducted on the formation of MSC-microcarrier aggregates and explored possible methods to overcome drawbacks associated with such culture strategies. It was suggested that hMSC aggregates generated from thermal responsive microcarriers in bioreactors maintained comparable immunomodulation and cytokine secretion compared to conventional culture strategies [62]. Heathman and co-workers reported a minimum agitation speed in a bioreactor system to obtain high cell numbers; however, low agitation were still accompanied by cell aggregation, leading to inconsistencies between pre- and post-harvest sampling. Therefore, an alternative oxygen supply method is needed to overcome the current downsides faced by readily available methods, which introduced shear forces to the cells during increased agitation speeds in up-scaling of cultures [63]. On the other hand, a protocol which utilised short periods of intense agitation in the presence of enzymes such that the cells were detached yet remained undamaged and retained post-harvest characteristics, was reported [54].

#### MSC differentiation

Aside from up-scaling MSC expansion, more researchers were shifting their focus towards inducing cell differentiation in microcarrier cultures simulating a three-dimensional human body environment. Lin et al. showed that chondrogenic pellets generated from microcarrier cultures developed larger pellet diameters, and produced more DNA, GAG and collagen II per pellet with greater GAG/DNA and collagen II/DNA ratios compared with that of tissue culture flasks, while similar result were observed by using another type of microcarrier [64]. An increasing number of studies have highlighted bone formation potential by using microcarrier cultures, for example, a new process developed by Zhang et al. fabricated pre-vascularized bone microtissues by integrating microcarrier culture and co-culture with MS and HUVEC [65]. Aside from that, Tanimowo et al. fabricated a novel agarose-k-casein microsphere which upregulated the expression of osteogenic differentiation markers in bone marrow MSCs [57]. A titanium phosphate glass microcarrier that enhances bone morphogenic protein-2 (BMP-2) and osteopontin (OPN) expression by h-MSC was introduced. BMP-2 is considered an important protein in cell differentiation and tissue regeneration, which is normally associated with osteoinductive growth factors [66]; OPN on the other hand is mainly related to bone metabolism and remodelling [67]. In this case, it was suggested that titanium phosphate glass microcarriers influenced hMSC differentiation and metabolic activity and could contribute in bone tissue engineering [55].

## Conclusion

Limitation of cell numbers in MSC-based cell therapy enlightened multiple approaches to increase the cell yield. Three-dimensional microcarrier cultures seems to be a potential candidate in the up-scaling production of MSCs. This review demonstrates that microcarriers, whether commercially available or produced in-house, were capable of enhancing production and inducing chondrogenic and osteogenic differentiation in MSCs.

However, several challenges in this system need to be addressed during cell manufacturing. The yields of MSC up-scale activity are still showing inconsistency from one another, even similar culture techniques and consumables were used. This problem could be possibly due to the batch-to-batch variances present in undefined media which relying on animal/human derived serum as main supplement. The variation from each batches of serum further affect the quality of the up-scaled product by different sources of origin, brands, and present of unidentified risk of contamination. In this case, one of the solutions to minimise this variations is the adaptation of serum free media (SFM) in MSC culture as mentioned by Ota et al. [68]. Aside from cell yield variations, the downstream harvesting approaches still require optimisation to improve cell recovery; in fact, MSC differentiation efficiency in 3D system remains uncertain and the mechanism is still not well-studied. Therefore, detailed studies are still required before this system to be adopted into the scale of GMP manufacturing.

## Data Availability

The datasets generated during and/or analysed during the current study are available in the Scopus, Ebscohost, and Pubmed repository.

## Abbreviations

MSC: Mesenchymal stem cell
GVHD: Graft versus host disease
PAMPs: Pathogen-associated molecular patterns
DAMPs: Damage-associated molecular patterns
BLM: Bleomycin
TNF: Tumor necrosis factor
IL: Interleukin
IFN: Interferon
APC: Antigen-presenting cell
GMP: Good manufacturing practice
DEAE: Diethylaminoethyl
PEG: Polyethylene glycol
HPL: Human platelet lysate
GAG: Glycosaminoglycan
